# Diagnosis of Tumours of the Central Nervous System

**Published:** 1946

**Authors:** Hugh Cairns

**Affiliations:** Nuffield Professor of Surgery, University of Oxford


### DIAGNOSIS OF TUMOURS OF THE CENTRAL NERVOUS SYSTEM

BY

SIR HUGH CAIRNS, D.M., F.R.C.S.

*Nuffield Professor of Surgery, University of Oxford.*

---

MY part in this discussion is to deal with the accessory methods of diagnosis, but first I feel that I must emphasize the importance of what Miss Beck has so ably claimed for clinical methods. Before using accessory methods the neurologist must assess the clinical picture and the patient. There are certain cases suspected of intracranial tumour in which thorough clinical investigation discloses the true nature of the condition—migraine, aural vertigo, anxiety states and so on : and in such cases the use of accessory methods may be not only unnecessary, but even harmful in heightening the patient's apprehension.

To use accessory methods effectively and in their correct order, the clinical picture must first be studied. In overt tumour syndromes, where there is advanced papilloedema and rise of intracranial pressure, lumbar puncture and ventriculography may be dangerous unless carried out as part of a carefully planned attack, with operation following hard upon the receipt of crucial diagnostic information supplied by the accessory methods. Furthermore, neurosurgery is no exception to the rule that in order to treat a patient satisfactorily it is necessary to know his background and to assess the degree and manner in which he has been disabled by his illness.

Having said so much, one can claim that, used judiciously, accessory methods may add remarkable precision to clinical neurological diagnosis, often revealing the precise situation of a tumour and sometimes providing important evidence as to its nature.

*Lumbar puncture.* The manometric pressure should always be measured, and, with the patient horizontal and relaxed, should normally be around 150 mm. of cerebrospinal fluid. Rise of pressure above 180 mm. is suspicious of tumour. In certain circumstances the protein content of the C.S.F. is important in pathological diagnosis, for with almost all intracranial tumours which are in contact with the large cerebrospinal spaces—the ventricles and the basal cisterns—the protein content of the C.S.F. is raised above 60 mgm. per cent. In brain-abscess also the protein in the C.S.F. is usually raised and in addition there is usually a moderate increase of the white cells, not necessarily all polymorphonuclears.

C

In the *surgery of the spinal cord* an advance has been made in recent years from increasing use of lumbar puncture and refinement of the Queckenstedt test, and the more liberal use of spinal radiography with opaque media. Any patient with progressive weakness of the legs should be promptly investigated by lumbar puncture, and, if the Queckenstedt test shows even a partial block, as indicated by slowing of the rise (or fall) of the C.S.F. in the manometer on (or after) jugular compression, there are good grounds for suspecting spinal tumour. In such cases opaque radiography on a tilting table will often reveal the situation of a spinal tumour before there is enough sensory disturbance to indicate the level of the tumour by clinical methods. Spinal tumours are usually benign, and when they can be removed at an early stage, the patient is spared months of incapacity while waiting for the sensory level to develop, and recovery is prompt and usually complete. In spinal radiography the substitution for lipiodol of myodil, which in this country was first made by Professor Wilson Baker at Oxford, has proved of value; for the fluid runs easily in the subarachnoid space, and appears to be quite innocuous.

In conclusion it must be emphasized that successful use of these accessory methods requires expensive apparatus, adequate technical assistance, and a considerable degree of technical skill on the part of the surgeon. The diagnostic precision obtained represents a considerable medical advance.

